# Metaphors We Think With: The Role of Metaphor in Reasoning

**DOI:** 10.1371/journal.pone.0016782

**Published:** 2011-02-23

**Authors:** Paul H. Thibodeau, Lera Boroditsky

**Affiliations:** Department of Psychology, Stanford University, Stanford, California, United States of America; Kyushu University, Japan

## Abstract

The way we talk about complex and abstract ideas is suffused with metaphor. In five experiments, we explore how these metaphors influence the way that we reason about complex issues and forage for further information about them. We find that even the subtlest instantiation of a metaphor (via a single word) can have a powerful influence over how people attempt to solve social problems like crime and how they gather information to make “well-informed” decisions. Interestingly, we find that the influence of the metaphorical framing effect is covert: people do not recognize metaphors as influential in their decisions; instead they point to more “substantive” (often numerical) information as the motivation for their problem-solving decision. Metaphors in language appear to instantiate frame-consistent knowledge structures and invite structurally consistent inferences. Far from being mere rhetorical flourishes, metaphors have profound influences on how we conceptualize and act with respect to important societal issues. We find that exposure to even a single metaphor can induce substantial differences in opinion about how to solve social problems: differences that are larger, for example, than pre-existing differences in opinion between Democrats and Republicans.

## Introduction

Both crime, and the criminal justice system designed to deal with crime, impose tremendous costs on society. Over 11 million serious crimes are reported in the United States each year [1], and the US has the highest per capita imprisonment rate of any country [2]. Despite being home to only 5% of the world's population, the United States holds 25% of the world's prisoners, with nearly 1% of the US population living behind bars [3]. Addressing the crime problem is an issue of central importance in social policy. How do people conceptualize crime, and how do they reason about solving the crime problem?

Public discourse about crime is saturated with metaphor. Increases in the prevalence of crime are described as *crime waves, surges* or *sprees*. A spreading crime problem is a *crime epidemic, plaguing a city* or *infecting a community*. Crimes themselves are *attacks* in which *criminals prey on unsuspecting victims*. And criminal investigations are *hunts* where criminals are *tracked* and *caught*. Such metaphorical language pervades not only discourse about crime, but nearly all talk about the abstract and complex [4]–[5]. Are such metaphors just fancy ways of talking, or do they have real consequences for how people reason about complex social problems like crime?

Previous work has demonstrated that using different metaphors can lead people to reason differently about notions like time, emotion, or electricity [6]–[11]. For example, people's reasoning about electricity flow differed systematically depending on the metaphoric frame used to describe electricity (flowing water vs. teeming crowds) [6]. Such findings on metaphorical framing are grounded in a larger body of work that has established the importance of linguistic framing in reasoning [12], and the importance of narrative structure in instantiating meaning [13]. However, questions about the pervasiveness of the role of metaphor in thinking remain. Critics argue that very little work has empirically demonstrated that metaphors in language influence how people think about and solve real-world problems [14].

In this paper we investigate the role of metaphor in reasoning about a domain of societal importance: social policy on crime. Beyond establishing whether metaphors play a role in how people reason about crime, our studies are designed to further illuminate the mechanisms through which metaphors can shape understanding and reasoning. If metaphors in language invite conceptual analogies, then different metaphors should bring to mind different knowledge structures and suggest different analogical inferences. In this paper we ask if metaphors indeed play such a role in reasoning about social policy. That is, do we reason about complex social issues in the same way that we talk about them: through a patchwork of metaphors?

Some observations of crime policy in the real world suggest that people may indeed take metaphors as more than just talk. For example, shifts in metaphors are often accompanied by shifts in policy. In the 1980s Ronald Reagan declared a *war on drugs*, with smugglers, dealers, and users defined as the enemy to be fought. Policies in line with the war on drugs mandated longer, harsher sentences for drug-related crime. Since then, the incarceration rate has more than quadrupled in the US [15].

Others have taken the *crime is a virus* metaphor seriously and have implemented programs to treat crime as a contagious disease. For example, a crime-prevention program run by an epidemiologist in Chicago treats crime according to the same regimen used for diseases like AIDS and tuberculoses, focusing on preventing spread from person to person [16].

Some criminal justice scholars have even implicated bad metaphor as the root of failure in crime prevention [17]. In one case described by Kelling, a serial rapist attacked 11 girls over a 15-month period before being captured by the police. During those 15 months, the police had information that (had they shared it with the community) could have prevented some of the attacks. Instead, they opted to keep that information secret to set traps for their suspect. The police, on Kelling's analysis, were entrenched in their metaphorical role of hunting down and catching the criminal, and neglected their responsibility to inoculate the community against further harm. The girls, Kelling writes, “were victims… not only of a rapist, but of a metaphor” (p. 1).

In this paper we empirically investigate whether using different metaphors to talk about crime indeed leads people to reason about crime differently and, in turn, leads them to propose different solutions to the crime problem. We will focus on two contrasting metaphors for crime: crime as a virus and crime as a beast. Do these metaphors subtly encourage people to reason about crime in a way that is consistent with the entailments of the metaphors? For example, might talking about crime as a virus lead people to propose treating the crime problem the same way as one would treat a literal virus epidemic? Might talking about crime as a beast lead people to propose dealing with a crime problem the same way as one would deal with a literal wild animal attack?

To help generate a clear set of predictions, we conducted a norming survey asking 28 participants on Amazon's Mechanical Turk (www.mturk.com; [18]) to describe what should be done to solve a literal virus or beast problem. We asked people to imagine a “virus infecting a city” or a “wild beast preying on a city” and then to describe the best way to solve the problem that they had imagined. Participants who imagined a “virus infecting the city” universally suggested investigating the source of the virus and implementing social reforms and prevention measures to decrease the spread of the virus. That is, they wanted to know where the virus was coming from, whether the city could develop a vaccine and how the virus was spreading. They also wanted to institute educational campaigns to inform residents about how to avoid or deal with the virus and encourage residents to follow better hygiene practices. Participants who imagined a “wild beast preying on a city” universally suggested capturing the beast and then killing or caging it. They wanted to organize a hunting party or hire animal control specialists to track down the beast and stop it from ravaging the city.

Might these schematic representations for solving literal virus or beast problems transfer to people's reasoning about crime if crime is metaphorically framed as a virus or a beast? That is, if crime is talked about as a virus, will people suggest diagnosing the root cause of the problem and enacting social reform to treat and inoculate the community? If crime is a beast, will people suggest catching and jailing criminals in order to fight off the crime attack?

In Experiment 1, we gave people a report about increasing crime rates in the City of Addison and asked them to propose a solution. For half of the participants, crime was metaphorically described as a beast preying on Addison, and for the other half as a virus infecting Addison. The rest of the report contained crime statistics that were identical for the two metaphor conditions. The results revealed that metaphors systematically influenced how people proposed solving Addison's crime problem. When crime was framed metaphorically as a virus, participants proposed investigating the root causes and treating the problem by enacting social reform to inoculate the community, with emphasis on eradicating poverty and improving education. When crime was framed metaphorically as a beast, participants proposed catching and jailing criminals and enacting harsher enforcement laws.

In Experiment 2, we modified the report and repeated the study. Whereas in Experiment 1, the metaphoric frame was established using vivid verbs with rich relational meaning in phrases scattered throughout the report (e.g., crime was said to be either preying & lurking, or infecting & plaguing). In Experiment 2, we used a single word to instantiate the metaphoric frame. Despite this small difference between the virus and beast conditions in the modified report (“Crime is a virus/beast ravaging the city of Addison”), we again found that participants in the two conditions offered different problem solving suggestions. The findings of Experiment 2 demonstrate that these relational elements need not be specified explicitly. People spontaneously extracted the relevant relational inferences even given a single metaphorical noun in Experiment 2.

In Experiment 3 we tested whether the influence of the metaphor observed in the first two studies could have come about through simple spreading activation from lexical associates of the words “beast” and “virus.” Perhaps simply hearing a word like beast, even outside of the context of crime, would activate representations of hunting and caging. These activated lexical associates might then bleed into people's descriptions of how to solve the crime problem. To test for this possibility we dissociated the words “beast” and “virus” from the metaphorical frame in Experiment 3. Before reading the crime report, participants were asked to provide a synonym to the word “beast” or the word “virus” – thereby priming representations for a beast or a virus. They then read the same report about crime as in Experiment 2, but with the metaphorical word omitted (“Crime is ravaging the city of Addison”). This disconnected lexical prime did not yield differences in people's crime-fighting suggestions, revealing that metaphors act as more than just isolated words – their power appears to come from participating in elaborated knowledge structures.

In Experiment 4 we tested whether metaphors can affect not only how people propose solving the problem of crime, but also how they go about gathering information for future problem solving. If participants seek out information that is likely to confirm the initial bias suggested by the metaphor, this may be a mechanism for metaphors to iteratively amass long-term effects on people's reasoning. Indeed, when people were presented with a metaphorically framed crime problem and then given the opportunity to gather further information about the issue, participants chose to look at information that was consistent with the metaphorical frame.

In Experiment 5 we investigated the time-course of how metaphors influence the construal of complex issues. One possibility is that metaphors influence reasoning by providing people a knowledge frame that structures subsequent information. After being exposed to the metaphor, participants assimilate all further information they receive into this knowledge structure, instantiating any ambiguous information in a way that would be consistent with the metaphor. If this is the case, if metaphors actively coerce incoming information, then metaphors should have the most impact when they are presented early. This was the structure of the report in Experiment 4 (and Experiment 2): the metaphoric frame was presented in the first sentence of the report.

Alternatively, if metaphors simply activate a stored package of ideas and do not encourage the kind of active assimilation process described above, then they should be most effective when they are presented late in the narrative, as close to when people are asked to reason about a solution as possible. This way, the memory of the metaphor should be fresh and any knowledge activated by it should have the best chance to influence reasoning. This was the structure of the report in Experiment 5: the metaphoric frame was presented in last sentence of the report. Unlike the results of Experiment 4, this late metaphorical framing had no effect on people's crime-related information foraging. These findings suggest that metaphors can gain power by coercing further incoming information to fit with the relational structure suggested by the metaphor.

One of the most interesting features of the effects of metaphor we find throughout these studies is that its power is covert. When given the opportunity to identify the most influential aspect of the crime report, participants (in all four studies that include a metaphoric frame) ignore the metaphor. Instead, they cite the crime statistics (which are the same in both conditions) as being influential in their reasoning. Together these studies suggest that unbeknownst to us, metaphors powerfully shape how we reason about social issues. Further, the studies help shed light on the mechanisms through which metaphors influence our reasoning.

## Methods

### Ethics Statement

The experiments reported here were done in accordance with the Declaration of Helsinki. Additionally, they followed the ethical requirements of the Stanford University institutional review board and complied with ethics guidelines set forth by the IRB recommendations. Participants were informed that their data would be treated anonymously and that they could terminate the experiment at any time without providing any reason. We received written informed consent from all participants before they participated in an experiment.

### Participants

In Experiment 1, 485 students – 126 from Stanford University and 359 from the University of California, Merced – participated in the study as part of a course requirement. Experiments 2–5 were conducted online with participants recruited from Amazon's mechanical Turk (347, 312, 185, and 190, respectively). In exchange for participation in the study, people were paid $1.60 – consistent with a $10/hour pay rate since the study took 5 to 6 minutes to complete.

Gathering data from these various sub-populations allowed us to sample a broader cross-section of the general population. This is important since people's conceptions of social issues like crime are likely to differ as a function of factors like socioeconomic status and personal experience. This is particularly true of the sample that was recruited online, which was more diverse than that available at Stanford specifically or on college campuses generally [18].

Running Experiments 2–5 online also afforded careful control over our sample population. We used Mechanical Turk's exclusion capabilities and tracked IP addresses to ensure that participants were not repeatedly sampled. We also restricted our study to Turkers with a 95% or better performance record to ensure that we were sampling high quality participants (“Requesters” have the opportunity to publicly give positive or negative feedback to their participants, which can then be used as a criterion for future “Requesters”). At the end of the online version of the study we asked participants to describe their language history, current geographic location, and provide some background information. We then restricted our analysis to residents of the United States who were native English speakers. The characteristics of our samples are detailed in the Results section below.

### Materials

In each of the five experiments, participants were presented with a survey that included a short paragraph about crime in the fictional city of Addison and some follow-up questions. The survey differed subtly between experiments, but always contrasted a crime-as-virus framing with a crime-as-beast framing.

It should be noted that there are two somewhat different metaphorical frameworks that treat crime as an illness. In one, the community or population is seen as an organism, and crime is a disease that is developing inside that organism (e.g., “Violent crime is a cancer that eats away at the very heart of society.”). In another, the community is seen as individual agents and crime is a contagious disease that can be passed on from one person to another forming an epidemic. In this paper the stimuli did not strongly distinguish between these different varieties of crime as illness metaphors, but doing so would be an interesting extension of this work, as these metaphors suggest somewhat different implications for treating crime.

#### Experiment 1

In the first experiment, participants were presented with one of two versions of the crime paragraph. The two versions of the paragraph differed only in the embedded metaphor: In one, crime was a beast; in the other, crime was a virus. The majority of the paragraph consisted of crime statistics, which were the same in both versions. Half of the participants were given the crime-as-beast version and half the crime-as-virus version. The paragraph read:

Crime is a {wild beast preying on/virus infecting} the city of Addison. The crime rate in the once peaceful city has steadily increased over the past three years. In fact, these days it seems that crime is {lurking in/plaguing} every neighborhood. In 2004, 46,177 crimes were reported compared to more than 55,000 reported in 2007. The rise in violent crime is particularly alarming. In 2004, there were 330 murders in the city, in 2007, there were over 500.

This report was followed up with two questions: 1) In your opinion what does Addison need to do to reduce crime? 2) Please underline the part of the report that was most influential in your decision. This question was aimed at discovering if participants explicitly noticed or made use of the metaphor.

#### Experiment 2

The crime report used in the second experiment was similar, but not identical to the one used in Experiment 1. Importantly, it instantiated the beast or virus metaphor for crime with a single word. It read as follows:

Crime is a {beast/virus} ravaging the city of Addison. Five years ago Addison was in good shape, with no obvious vulnerabilities. Unfortunately, in the past five years the city's defense systems have weakened, and the city has succumbed to crime. Today, there are more than 55,000 criminal incidents a year - up by more than 10,000 per year. There is a worry that if the city does not regain its strength soon, even more serious problems may start to develop.

In Experiment 2, we asked three follow-up questions in the following order: 1) In your opinion what does Addison need to do to reduce crime? 2) What is the role of a police officer in Addison? 3) Please copy the part of the report that was most influential and paste it in the text area below. Questions one and two were free-response. Question three was copy and paste (participants were shown the report adjacent to an open text field and were asked to copy the portion of the report that was most influential in their reasoning and paste it into the open text field).

#### Experiment 3

The design of Experiment 3 was similar to that of Experiment 2; however, before participants read the crime report, they were shown the word “beast” or the word “virus” and were asked to “list a synonym” for it. After completing this task, they were presented with the paragraph on crime in Addison on a separate screen. The crime report used in Experiment 3 was the same as the crime report for Experiment 2, except that it did not contain a virus or beast metaphor. The first sentence of the report read: “Crime is ravaging the city of Addison.” It was otherwise identical to the report from Experiment 2.

#### Experiment 4

The crime report used in Experiment 4 was the same as the crime report used for Experiment 2. However, instead of asking the follow-up questions from Experiments 2 and 3, we asked participants to select one of four crime-related issues for further investigation – with the knowledge that this information should be used to help them make a more informed crime-reducing suggestion. The instructions read as follows: “Now imagine that Addison has consulted you about the crime problem. You have the resources to investigate one of the following four issues. Please select one from the list below.” The issues included: 1) the education system and availability of youth programs, 2) the economic system including the poverty level and employment rate, 3) the size and charge of the police force, and 4) the correctional facilities including the methods by which convicted criminals are punished.

#### Experiment 5

The materials and task in Experiment 5 were identical to those of Experiment 4 except, instead of presenting the metaphor frame at the beginning of the report, we presented the metaphor frame at the end of the report, as shown below. All other aspects of the design were identical to Experiment 4. The paragraphs used were:

Five years ago Addison was in good shape, with no obvious vulnerabilities. Unfortunately, in the past five years the city's defense systems have weakened, and the city has succumbed to crime. Today, there are more than 55,000 criminal incidents a year - up by more than 10,000 per year. There is a worry that if the city does not regain its strength soon, even more serious problems may start to develop. Crime is a {beast/virus} ravaging the city of Addison.

### Design

In Experiment 1 the survey was included in a larger packet of questionnaires that were unrelated to this study.

In Experiments 2–5, each step of the experiment was presented on a separate screen. That is, the initial crime report was presented on a screen by itself. After participants read the report and clicked a button indicating they had finished reading it, the report disappeared and the first follow-up question appeared on a screen by itself. Similarly, each subsequent question was shown on a separate screen. On the final screen, participants were asked several background questions (e.g., What is the first language you learned to speak?).

Participants in Experiments 2–5 were explicitly instructed not to use the “back” button on their browser. If they did use the “back” button, the experimental session was terminated. This ensured that participants did not reread the crime report when they were later asked questions about it.

## Results

### Experiment 1

In Experiment 1, we explored whether framing a crime problem with one of two contrasting metaphors for crime could systematically influence how people reasoned about the problem. Participants were presented with one of two versions of the crime paragraph (as detailed above) and asked a set of free response follow-up questions. Of particular interest, participants were asked how they would recommend solving Addison's crime problem.

#### Coding

Proposed solutions to the crime problem in Addison were coded into two categories in line with the results of the norming study described in the introduction: 1) diagnose/treat/inoculate, and 2) capture/enforce/punish. Responses were categorized as “diagnose/treat/inoculate” if they suggested investigating the underlying cause of the problem (e.g., “look for the root cause”) or suggested a particular social reform to treat or inoculate the community (e.g., fix the economy, improve education, provide healthcare). Responses were categorized as “capture/enforce/punish” if they focused on the police force or other methods of law enforcement (e.g., calling in the National Guard) or modifying the criminal justice system (e.g., instituting harsher penalties, building more jails). For brevity, we will refer to the “diagnose/treat/inoculate” category as “reform” and the “capture/enforce/punish” category as “enforce.”

Each participant's response was weighted equally – as a single point towards the analysis. For solutions that solely emphasized either reform or enforcement, the respective category was incremented by a point. Responses that exclusively emphasized one approach were the majority. Occasionally, however, participants listed both types of suggestions. In this case, if the response listed a disproportionate number of suggestions that were consistent with one approach (e.g., if the response listed three suggestions in line with reform and only one in line with enforcement, as in “investigate the root cause, institute new educational programs, create jobs, and hire more police”) then it was coded as a full point for the corresponding category. However, if the response equally emphasized both approaches, then the point was split between the categories such that each was incremented by .5.

Thirty of the 485 responses (6%) did not fit into either category. In every case this was because the response lacked a suggestion (e.g., “I don't know”, “I need more information”, “It should be addressed”). These data were omitted from analysis.

Participants' crime reducing suggestions were coded blindly by two coders. Cohen's kappa – a measure of inter-rater reliability – was .75 indicating good agreement between the coders (*p*<.001). All disagreements between the coders were resolved between them before analyzing the data.

#### Results

**Figure 1:**
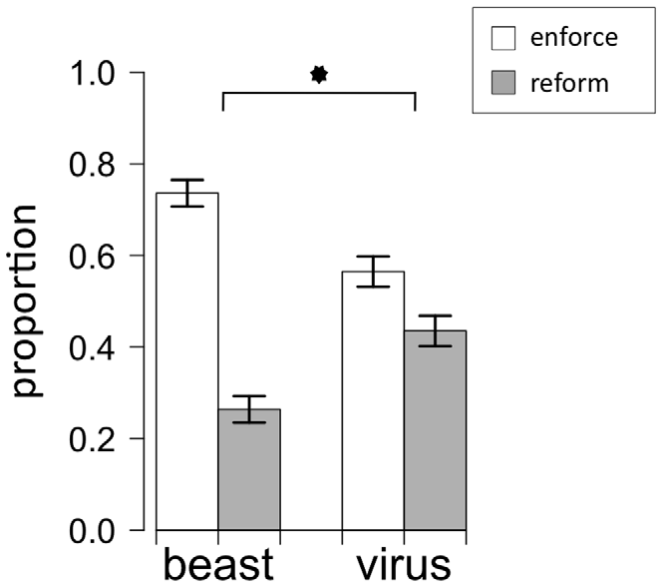
Proportion of proposed solutions to crime by metaphor frame.

Overall, participants were more likely to emphasize enforcement strategies (65%) than reform (35%), *χ* ^2^ = 41.85, *p*<.001. However, as predicted, the solutions participants proposed to the crime problem in Addison differed systematically as a function of the metaphorical frame encountered in the crime report (see Fig. 1). Participants given the crime-as-beast metaphorical framing were more likely to suggest enforcement (74%) than participants given the crime-as-virus framing (56%), *χ* ^2^ = 13.94, *p*<.001. See Table 1 for response frequencies.

Interestingly, when asked to identify the most influential aspect of the report, most participants ignored the metaphor. Only 15 participants (3%) identified the metaphoric frame as influential to their problem solving strategy. Removing these participants from the analysis did not affect the results (the proportion of responses that were congruent with the metaphor was not different in the two analyses, *χ* ^2^ = .0001, *p* = .991). The vast majority of the participants identified the statistics in the crime report as being most influential in their decision – namely, the final three sentences of the paragraph that state the increasing crime and murder rate.

**Table 1:** Response frequencies for each of the five experiments by condition and response category.

EXPERIMENT	1		2		3		4		5	
CONDITION	beast	virus	beast	virus	beast	virus	beast	virus	beast	virus
**ENFORCE**	170	126.5	80	72	75	66	33	21	27	30
**SOCIAL**	61	97.5	33	61	43	36	50	74	61	54

#### Discussion

In this experiment, we found that crime-reducing suggestions differed systematically as a function of the metaphor used to frame the crime problem. Participants who read that crime was a virus were more likely to propose treating the crime problem by investigating the root causes of the issue and instituting social reforms than participants who read that crime was a beast. Participants who read that crime was a beast were more likely to propose fighting back against the crime problem by hiring police officers and building jails – to catch and cage the criminals – than participants who read that crime was a virus.

Further, despite the clear influence of the metaphor, we found that participants generally identified the crime statistics, which were the same for both groups, and not the metaphor, as the most influential aspect of the report. These findings suggest that metaphors can influence how people conceptualize and in turn approach solving an important social issue, even if people don't explicitly perceive the metaphor as being especially influential.

### Experiment 2

In Experiment 2, we made two substantive changes to the task to further test the role of metaphor in reasoning. First, we changed how the metaphoric frame was presented. In Experiment 1, the metaphoric frame was established several times and included vivid relational language. For example, crime was said to be either preying & lurking, or infecting & plaguing the community. These metaphorical verbs explicitly specified relations between crime and the community. Is specifying relations explicitly in this way necessary for people to make appropriate inferences, or might people be able to spontaneously extract the relevant relational inferences given a minimal metaphorical suggestion? Might a single carefully chosen and appropriately placed word be enough to instantiate a metaphorical frame and induce different reasoning strategies?

In Experiment 2 we tested this hypothesis by removing the relational verbs from the report. We replaced them with a single word metaphor that described crime as a “virus” or “beast” in the introductory sentence. The two conditions differed only in this one word, and otherwise included all the same information.

The second change we made was that we added an additional follow-up question: What is the role of a police officer in Addison? This question aimed to disambiguate the modal crime-reducing suggestion from Experiment 1, which was “increase the police force.” In that context, we interpreted the response (and close variants of it) as a suggestion for increased law enforcement and punishment. However, police officers do not just catch and punish criminals. They also serve as crime deterrents, educators, and role models and it is possible that some participants intended for the increased police presence to serve in this way. Including this question allowed these participants an opportunity to explicitly specify how they envisioned the increased police force impacting the community.

#### Participant characteristics

We restricted our analysis of the initial sample of 347 Turkers to residents of the United States who were native English speakers. This left data from 253 participants for analysis (i.e., 94 participants were excluded – 27% of the initial dataset). Of these 253 participants, 157 were female and 96 were male. Their ages ranged from 18 to 66, with a mean age of 32 (and median age of 29). Eighty-two reported an affiliation to the Democratic Party, 57 reported an affiliation to the Republican Party, and 114 were Independent.

#### Coding

Crime-reducing suggestions were coded into two groups (reform and enforcement) as they were in Experiment 1. However, in Experiment 2 we coded one additional feature of this question: whether the participant exclusively suggested increasing the police force. For these responses, we planned to use the follow-up question about the role of a police officer in Addison to disambiguate whether the participant thought a police officer's primary role was as an instrument of social reform and prevention or an instrument of law enforcement and punishment.

Interpretations of the role of a police officer were coded into two groups that were analogous to the categories created for the first question: 1) crime deterrent, and 2) law enforcer and punisher. Interpretations that emphasized the police officer's role in preventing crime, educating youth, or serving as a role model in the community were coded as “crime deterrent.” Interpretations that emphasized the police officer's role in catching criminals, responding to crime reports, or punishing criminals were coded as “law enforcer and punisher.” As in Experiment 1, each response contributed one point to the analysis. This point either went entirely to one of the two categories or was split evenly between them.

Seven (3%) crime-reducing suggestions and 18 (7%) police officer interpretations were not coded. In every case this was because the response lacked a suggestion or interpretation and were eliminated from the analysis. It is possible that relatively more police officer interpretations fell into this category because the question was not prefaced with “In your opinion” (several responses to this question were a variant of “the report didn't say what the role of a police officer in Addison was”).

Answers to both of the free response questions were coded blindly by two coders. Inter-rater reliability was high for both: Cohen's kappa for crime-reducing suggestions was .86 (*p*<.001); Cohen's kappa for interpretations of the role of a police officer was .72 (*p*<.001). All disagreements between the coders were resolved between them before analyzing the data.

#### Results

The results of Experiment 2 replicate our findings from Experiment 1. Participants were again overall more likely to suggest enforcement (62%) than reform (38%), χ^2^ = 13.67, p<.01. However, the tendency towards enforcement was more pronounced among participants who read that crime was a beast (71%) than among participants who read that crime was a virus (54%), χ^2^ = 6.50, p<.05. See Table 1 for response frequencies by condition.

**Figure 2:**
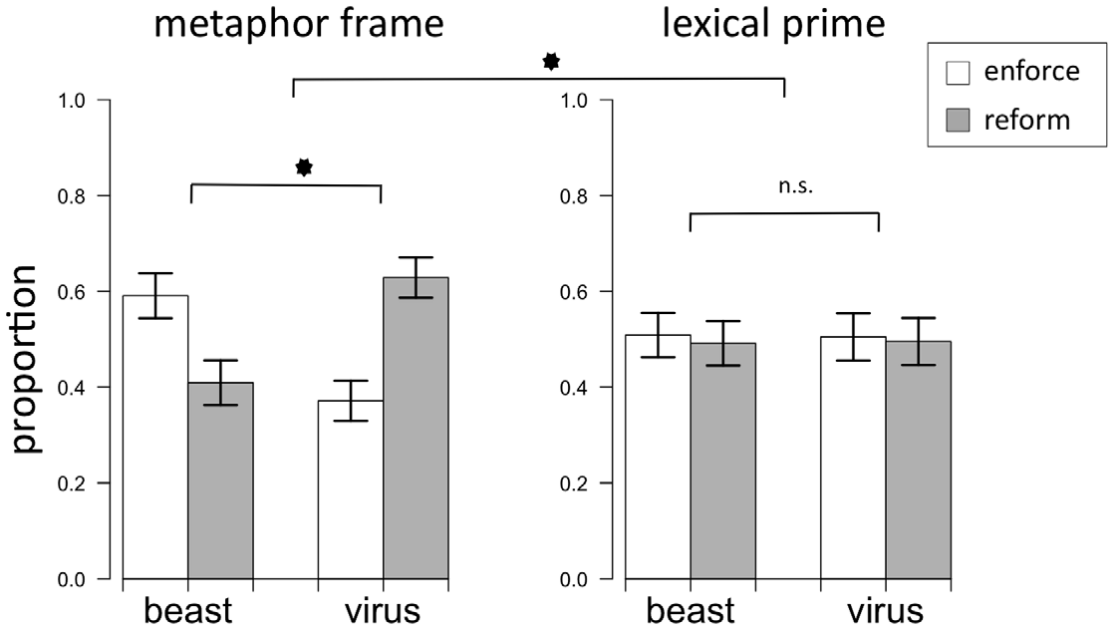
Proportions of responses to “solve the crime problem in Addison” (with “increase police” disambiguated). The left panel displays results from Experiment 2 (with a one-word metaphor frame); the right panel displays results from Experiment 3 (in which a synonyms task preceded the non-metaphorically framed paragraph).

Of the responses, 81 (31%) exclusively suggested increasing the police force. Disambiguating these responses by the participants' corresponding views of the role of a police officer in Addison further clarified the effect of the metaphor. Because “police” responses were previously coded as enforcement, disambiguating them created an overall shift to the reform category in both conditions, with a larger shift in the virus condition as predicted. With the “police” responses disambiguated, 37% of the responses advocated enforcement in the virus condition, and 59% advocated enforcement in the beast condition, χ^2^ = 10.76, p<.01 (see Fig. 2).

Further, as in Experiment 1, participants did not explicitly report the metaphor as being influential in their reasoning. Only 18 of the 253 participants (7%) identified the metaphor as influential. Excluding participants who identified the metaphor as influential did not change the reported results (the proportion of responses that were congruent with the metaphor was not different in the two analyses, χ^2^ = .01, p = .92).

#### Discussion

Results of Experiment 2 replicate and extend the findings of Experiment 1. Manipulating the metaphor used to frame the issue of crime influenced how people approached solving the crime problem. When crime was framed as a virus, participants were more likely to suggest social reform. Alternatively, when crime was framed as a beast, participants were more likely to suggest law enforcement and punishment.

Remarkably, presenting an otherwise identical report with only one word different in the introductory frame (“Crime is a virus/beast ravaging the city of Addison”) yielded systematically different problem solving suggestions just as in Experiment 1. While in Experiment 1, the metaphoric frame was established using vivid verbs with rich relational meaning (e.g., crime was said to be either preying & lurking, or infecting & plaguing). The findings of Experiment 2 demonstrate that these relational elements need not be specified explicitly. People spontaneously instantiated the relevant relational inferences even given a single metaphorical noun in Experiment 2.

Further, in Experiment 2 we asked participants to provide their views on the role that a police officer should play in Addison. This afforded us a clearer interpretation of their crime-reducing suggestions and boosted our power to detect the influence of the metaphor.

Interestingly, despite the clear influence of the metaphor, we found that participants generally identified the crime statistics, which were the same for both groups, and not the metaphor, as the most influential aspect of the report.

### Experiment 3

In Experiment 3 we tested whether the influence of the metaphor observed in the first two studies could have come about through simple spreading activation from lexical associates of the words “beast” and “virus.” Perhaps simply hearing a word like beast, even outside of the context of crime, would activate lexical associates like “hunting” and “caging”. These activated lexical associates might then color people's descriptions of how to solve the crime problem. To test for this possibility we dissociated the words “beast” and “virus” from the rest of the crime report in Experiment 3. Before reading the crime report, participants were asked to provide a synonym to the word “beast” or the word “virus” – thereby priming representations for a beast or a virus. They then read the same report about crime as in Experiment 2, but with the metaphorical word omitted (“Crime is ravaging the city of Addison”). Might a non-metaphorical lexical prime have the same effect as a metaphor?

#### Participant characteristics

Of the 312 Turkers that were initially sampled for Experiment 2, 76 (24%) were excluded because they did not live in the United States or because they were not native English speakers. This left data from 236 participants for analysis. Of these 236 participants, 136 were female and 100 were male. Their ages ranged from 18 to 81, with a mean age of 29 (and median age of 26). Seventy-six reported an affiliation to the Democratic Party, 48 reported an affiliation to the Republican Party, and 112 were Independent.

#### Coding

Answers to the free response questions were coded as they were in Experiment 2. Fifteen crime-reduction suggestions (6%) and 21 police officer interpretations (9%) did not fit into either category. In every case this was because the response lacked a suggestion or interpretation.

Answers to both of the free response questions were coded blindly by two coders. Inter-rater reliability was high for both: Cohen's kappa for crime-reducing suggestions was .87 (*p*<.001); Cohen's kappa for interpretations of the role of a police officer was .84 (*p*<.001). All disagreements between the coders were resolved between them before analyzing the data.

#### Results

The synonyms that participants listed were analyzed to ensure that the lexical prime had the intended effect. Of the 124 participants in the crime-as-beast condition, all except one listed a synonym of “beast”. The modal response was “animal”, but others included “monster”, “mongrel”, “invader”, etc. The single respondent who did not list a synonym to “beast” instead wrote “I forget what a synonym is.” This participant's subsequent responses were omitted from the analyses reported below. Of the 112 participants in the crime-as-virus condition, all listed a synonym of virus. In this case, the modal response was “disease”, but others included “bug”, “cold”, “sickness”, “illness”, etc.

In Experiment 3, unlike Experiments 1 and 2, there was no difference in crime-reducing suggestions as a function of the condition – i.e., whether the participant listed a synonym to “virus” or “beast” before reading the crime paragraph did not affect what solutions they suggested to the crime problem. Overall, participants were significantly more likely to suggest enforcement or punishment (64%) than social reform (36%), *χ* ^2^ = 18.0, *p*<.001; however, there was no difference between participants who were lexically primed with “beast” (64% suggesting enforcement and punishment) versus those who were lexically primed with “virus” (65%), *χ* ^2^ = .001, *p* = .99. See Table 1 for response frequencies by condition.

Further, disambiguating the responses that called for an increase to the police force did not differentiate the groups. Sixty-eight of the 235 responses (29%) were disambiguated. Of these, 29 (43%) interpreted the role of a police officer as a crime deterrent, 37 (54%) interpreted the role of a police officer as a law enforcer or punisher, and two responses could not be disambiguated. This disambiguation did not reveal a difference between conditions: Participants who were lexically primed with “virus” were no more likely to suggest enforcement (50%) than those who were lexically primed with “beast” (51%), *χ* ^2^ = .006, *p* = .94 (see Fig. 2).

Comparing the results from Experiments 2 and 3 we find an interaction between the form in which the word “beast” or “virus” is presented (i.e., metaphor vs. lexical prime) and the extent to which crime-reducing suggestions are congruent with the prime. That is, we find that the metaphor in Experiment 2 was significantly more influential than the lexical prime in Experiment 3. To quantitatively compare the results of the two experiments we performed a chi-square contingency test as well as a set of logistic regressions. In Experiment 2, 61% of the responses were congruent with the metaphor (i.e., suggested “reform” when presented with crime-as-a-virus or suggested “enforcement” when presented with crime-as-a-beast), whereas only 50% of the responses in Experiment 3 were congruent with the lexical prime, *χ* ^2^ = 4.23, *p*<.05. Similarly, a logistic regression revealed that an interaction term for *experiment X condition* was a significant predictor of people's crime-fighting suggestions: a model that included the three predictors (experiment, condition, and the interaction term) was significantly better than a model with two predictors (omitting the interaction term), *χ* ^2^ (1, 459) = 5.85, *p<*.05.

#### Discussion

In Experiment 3 we tested whether the influence of the metaphor observed in the first two studies could have come about through simple spreading activation from lexical associates of the words “beast” and “virus.” We dissociated the words “beast” and “virus” from the story, so that they could act as non-metaphorical lexical primes. These disconnected lexical primes did not yield differences in people's crime-fighting suggestions. These results suggest that metaphors act as more than just isolated words – their power appears to come from participating in elaborated knowledge structures.

Additionally, the results of Experiment 3 shed some light on this population's baseline preference for reducing crime. That is, in Experiment 2 it might have been the case that participants had a general preference for reducing crime through enforcement and that it was the crime-as-virus frame alone that shifted peoples' responses. The results of Experiment 3, however, suggest that the population does not seem to favor either of the two crime-reducing suggestions absent a metaphoric frame and that both frames are influential.

### Experiment 4

In Experiment 4 we tested whether the influence of the metaphor would persevere even if people were able to select responses from a full set of options. One possibility is that a metaphorical frame affects what kind of solution comes to mind easiest. However, when faced with a complete set of options, people may realize they had neglected to attend to other alternatives and no longer show the influence of the metaphor. For example, a participant in the “beast” frame may not have spontaneously thought to address underlying problems in the economy or education. However, if these are made explicitly available as response options, the participant may recognize them as good ideas and may re-bound from the metaphorical framing. To test for this, in Experiment 4, we presented participants with a list of four possible approaches to the crime problem and asked them to choose one. These included two options that were more consistent with social reform (education, economy) and two options that were more consistent with enforcement and punishment (police, jails).

Rather than asking participants to make a crime-reducing suggestion as in previous studies, the task in Experiment 4 was to select an area to investigate further (in preparation to making a crime-fighting suggestion). This aspect of the experiment was designed to test whether metaphors can affect not only how people propose solving the problem of crime, but also how they go about gathering information for future problem solving. If participants seek out information that is likely to confirm the initial bias suggested by the metaphor, this may be a mechanism for metaphors to iteratively amass long-term effects on people's reasoning (as people seek out more and more confirming evidence).

#### Participant characteristics

Of the 185 Turkers who participated in Experiment 4, seven (4%) were excluded because they did not live in the United States or because they were non-native English speakers. This left data from 178 participants for analysis. Of these 178 participants, 89 were female and 89 were male. Their ages ranged from 18 to 70, with a mean age of 31 (and median age of 28). Seventy-eight reported an affiliation to the Democratic Party, 28 reported an affiliation to the Republican Party, and 72 were Independent.

#### Coding

Choosing to gather additional information about the education system or economic system was coded as a social reform category of response; gathering additional information about the police force or criminal justice system was coded as an enforcement and punishment category of response.

#### Results

Results of Experiment 4 replicate the effects of metaphorical frames found in Experiments 1 and 2. Participants who were presented with the crime-as-a-beast metaphor were more likely to gather additional information about the city's criminal justice system (40%) than participants who were presented with the crime-as-a-virus metaphor (22%), *χ* ^2^ = 5.72, *p*<.05. See Table 1 for response frequencies by condition.

As we saw in Experiments 1 and 2, when given the opportunity to identify the most influential aspect of the report, the vast majority ignored the metaphor. Only 27 participants (15%) reported that the metaphor influenced their decision. Eliminating these participants from the analysis does not change the results (the proportion of responses that were congruent with the metaphor was not different in the two analyses, *χ* ^2^ = .003, *p* = .96).

#### Discussion

In Experiment 4 we found that the effect of metaphorical framing persists even when the list of all possible approaches to solving crime is explicitly presented. Laying out four possible approaches to crime shifted the overall likelihood that people wanted to pursue social reform. It seems that explicitly seeing the space of possible responses makes people more likely to attempt reducing crime through reform than enforcement. However, we still found that peoples' responses were influenced by the frame that they read. Additionally, the results of Experiment 4 reveal that the metaphorical frame influences how people go about gathering information for future problem solving. People tended to seek additional information about the city that confirmed their initial (metaphor-induced) suspicion about how to solve crime.

### Experiment 5

In Experiment 5 we investigated the time-course of how metaphors influence people's construal of and reasoning about problems. One possibility is that metaphors influence reasoning by instantiating a knowledge frame that structures subsequent information. After being exposed to the metaphor, participants may assimilate all further information they receive into this knowledge structure, instantiating any ambiguous information in a way that would be consistent with the metaphor. For example, words like “vulnerabilities”, “defense”, “weakened” may take on different meanings depending on whether they are understood in the context of viruses or beasts [13], [19]. If this is the case, if metaphors actively coerce incoming information, then metaphors should have the most impact when they are presented early, such that their impact can accumulate in the course of assimilating further information.

Alternatively, if metaphors simply activate a fossilized package of ideas and do not encourage the kind of assimilation process described above, then they should be most effective when they are presented late in the narrative, as close to when people are asked to reason about a solution as possible. This way, the memory of the metaphor should be fresh and any knowledge activated by it should have the best chance to influence reasoning. In Experiment 5, we repeated the design of Experiment 4, but moved the metaphorical frame so that instead of being the first sentence in the crime report it was the last.

#### Coding

As in Experiment 4, choosing to gather additional information about the education system or economic system was coded as a social reform category of response; gathering additional information about the police force or criminal justice system was coded as an enforcement and punishment category of response.

#### Results

As in Experiment 4, participants in Experiment 5 were overall more likely to gather information relating to the city's social situation (67%) than the criminal justice system (33%), *χ* ^2^ = 19.55, *p*<.001.

**Figure 3:**
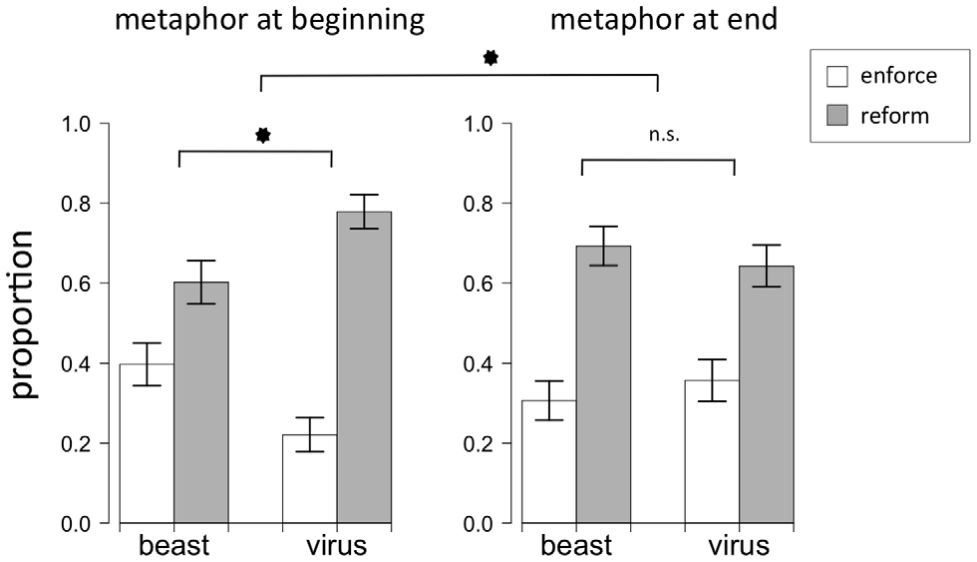
Seeking additional information. The left panel displays results from Experiment 4 (with a one-word metaphor frame at the beginning of the report); the right panel displays results from Experiment 5 (with the same one-word frame but at the end of the report).

However, unlike Experiment 4, there was no effect of the metaphorical frame. Participants who were presented with the crime-as-a-beast metaphor were about equally likely to gather additional information about the city's social situation (69%) as participants who were presented with the crime-as-a-virus metaphor (64%), *χ* ^2^ = .29, *p* = .59 (see Fig. 3). See Table 1 for response frequencies by condition.

This pattern was significantly different from the effects found in Experiment 4, *χ* ^2^ = 5.45, *p*<.05. That is, significantly more participants were influenced by the metaphor when it was presented at the beginning of the report (Experiment 4) than at the end of the report (Experiment 5). This conclusion is also supported by a logistic regression, which revealed that an interaction term for *experiment X condition* was a significant predictor of people's crime-fighting suggestions: a model that included the three predictors (experiment, condition, and the interaction term) was significantly better than a model with two predictors (omitting the interaction term), *χ* ^2^ (1, 346) = 5.34, *p<*.05.

As we saw in the previous experiments, when given the opportunity to identify the most influential aspect of the report, the vast majority ignored the metaphor. Only 18 participants (10%) reported that the metaphor influenced their decision.

#### Discussion

In Experiment 5 we investigated whether when a metaphor is introduced affects the metaphor's influence. Experiment 5 repeated the design of Experiment 4, but we moved the metaphorical frame so that instead of being the first sentence in the crime report it was the last. Unlike the results of Experiment 4, this late metaphorical framing had no effect on people's crime-related information foraging. These findings suggest that metaphors can gain power by coercing further incoming information to fit with the relational structure suggested by the metaphor.

These results are particularly striking since in Experiment 5, the metaphorical frame appears in much closer proximity to the measure of interest. It would have been reasonable to predict that a metaphorical frame that is more fresh in mind should have the largest effect. Instead, the way a metaphorical frame is integrated into the narrative appears to be more important. This finding also helps allay a possible worry about the findings in Experiment 3. In Experiment 3, we moved the words “virus” and ”beast” out of the crime story, and asked participants to generate synonyms to these words before they read about crime. When the words appeared in this way as disconnected lexical primes, they had no influence over people's crime-fighting suggestions. Of course, one possibility is simply that taking the words out of the narrative also made them more distant in time from the measure of interest. Results of Experiment 5 suggest that it is integration at the right point in the narrative rather than simple temporal distance that modulates our effects. In Experiment 5, the words “virus” and “beast” occurred immediately prior to the measure of interest, and yet had no effect.

## Discussion

In five experiments we investigated the role of metaphor in guiding how people reason about the complex problem of crime. We found that metaphors exert an influence over people's reasoning by instantiating frame-consistent knowledge structures, and inviting structurally-consistent inferences. Further, when asked to seek out more information to inform their decisions, we found that people chose information that was likely to confirm and elaborate the bias suggested by the metaphor – an effect that persisted even when people were presented with a full set of possible solutions.

Our results suggest that even fleeting and seemingly unnoticed metaphors in natural language can instantiate complex knowledge structures and influence people's reasoning in a way that is similar to the role that schemas [20], [21], scripts [22], [23], and frames [24] have been argued to play in reasoning and memory [13], [25]–[27]. That is, the metaphors provided our participants with a structured framework for understanding crime in Addison, influenced the inferences that they made about the crime problem, and suggested different causal interventions for solving the problem. This was true even though the metaphors themselves did not strike our participants as particularly influential.

Consistent with previous work on meaning instantiation, we find that the metaphors were most effective when they were presented early in the narrative and were then able to help organize and coerce further incoming information. For example, Bransford and Johnson demonstrate that a procedural description of washing clothes was understood and remembered best when participants knew the topic of the passage before they heard the description [13]. When the topic was given at the end of the passage or not at all, participants reported being unable to make sense of what they had heard and were able to recall few details of the description on a memory test. While the crime passage we used was clearly not as ambiguous as the procedural description of washing clothes used by Bransford and Johnson, it did contain many words and phrases that would likely be interpreted differently in the different contexts represented by the metaphoric frames. For instance, in the context of an attacking beast the meaning of the words “vulnerable” and “defense system” may be different from what the same words would be taken to mean in the context of a spreading virus. Previous work has demonstrated that contextual cues can strongly influence how people interpret seemingly unambiguous text [19], [28]–[29].

A further question is how such knowledge structures for thinking about crime emerge? How do people build virus-like or beast-like representations of crime and what is the role of linguistic metaphor in encouraging the construction of such knowledge structures? One potential mechanism is offered by work in analogical reasoning [6], [30]–[35]. For example, Bowdle & Gentner suggest that metaphors when first encountered are processed as analogies or structural alignments [35]. When we first hear about crime described as a beast, for example, we may carry out comparisons to discover any alignable similarities between crime and beasts. If such similarities are discovered, they can license the transfer of inferences from one domain to the other, and the most striking or stable structural similarities can be highlighted and stored in memory. With exposure to the system of “beast” metaphors, an elaborated knowledge structure can emerge for thinking about crime that mirrors in important relational structure the representations we have about the behavior of wild beasts. Through analogical transfer in this way, systems of metaphors in language can encourage the creation of systems of knowledge in a wide range of domains. Our reasoning about many complex domains then can be mediated through these patchworks of analogically-created representations.

A final question is how strong the influence of metaphorical framing really is? Focusing on a real-world social issue like crime allows us to compare the effects of metaphor we observe in the lab with the opinion differences that exist naturally in the population. People with different political affiliations hold different opinions on how to address societal problems like crime. How do the differences we find between metaphorical conditions compare to those between Democrats and Republicans, for example?

At the end of Experiments 2–5, we asked participants to report their political affiliation (Democrat, Independent, or Republican) and their gender. We found a predictable relationship between political affiliation and the tendency to emphasize enforcement in one's response. Across the four experiments, 48% of responses from Republicans emphasized enforcement whereas only 40% of responses from Democrats and Independents emphasized enforcement (data from Democrats and Independents did not differ from one another and so were collapsed). A logistic regression revealed political affiliation to be a significant predictor of people's crime-fighting suggestions: comparing a model with political affiliation included as a predictor to a constant-only model was statistically significant, *χ* ^2^ (1, 839) = 3.98, *p<*.05. We also found systematic differences by gender: 46% of responses from men and 38% of responses from women suggested enforcement. Comparing a logistic regression model with gender included as a predictor to a constant-only model was statistically significant, *χ* ^2^ (1, 839) = 5.389, *p<*.05.

Impressively the differences in opinion generated by the metaphorical frames were larger than those that exist between Democrats and Republicans, or between men and women. Metaphorical frames caused shifts of 18–22% in enforcement responses in Experiments 2 and 4. Differences between people of different political affiliations or between the two genders were 8–9%. To statistically compare the strength of these different predictors, we fit a set of logistic regression models for data from Experiments 2 and 4. We found that a model fit with a predictor for metaphor frame was significantly better than a constant-only model, *χ* ^2^ (1, 839) = 17.35, *p<*.001; however, including a predictor for gender, *χ* ^2^ (1, 839) = 0.013, *ns*, or political affiliation, *χ* ^2^ (1, 839) = 2.06, *ns*, or both, *χ* ^2^ (3, 839) = 3.03, *ns*, did not improve the model significantly. This analysis reveals a striking effect of metaphor as measured against real-world differences in opinion that exist in the population and impact policy-making.

Interestingly, we found that self-identified Republicans were also less likely to be influenced by the metaphors than were Democrats and Independents. Looking at data from Experiments 2 and 4 we find that 63% of the responses from Democrats and Independents are congruent with the metaphorical frame, whereas only 49% of those from Republicans were congruent with the metaphor. A logistic regression revealed that political affiliation was indeed a significant predictor of congruence with the metaphorical frame: comparing a model with political affiliation as a predictor against a constant-only model was statistically significant, *χ* ^2^ (1, 839) = 5.46, *p<.05*. These results may be consistent with previous analyses showing a difference in openness between people of different political affiliations [36]. Men and women were equally influenced by the metaphorical frames.

The studies presented in this paper demonstrate that even minimal (one-word) metaphors can significantly shift people's representations and reasoning about important real-world domains. These findings suggest that people don't have a single integrated representation of complex issues like crime, but rather rely on a patchwork of (sometimes disconnected or inconsistent) representations and can (without realizing it) dynamically shift between them when cued in context.

Metaphor is incredibly pervasive in everyday discourse. By some estimates, English speakers produce one unique metaphor for every 25 words that they utter [37]. Metaphor is clearly not just an ornamental flourish, but a fundamental part of the language system [28], [38]. This is particularly true in discussions of social policy [5], [39]–[40], where it often seems impossible to “literally” discuss immigration, the economy, or crime. If metaphors routinely influence how we make inferences and gather information about the social problems that confront us, then the metaphors in our linguistic system may be offering a unique window onto how we construct knowledge and reason about complex issues.

### Conclusions

The way we talk about complex and abstract ideas is suffused with metaphor. In five experiments, we have explored how these metaphors influence the way that we reason about complex issues and forage for further information about them. We find that metaphors can have a powerful influence over how people attempt to solve complex problems and how they gather more information to make “well-informed” decisions. Our findings shed further light on the mechanisms through which metaphors exert their influence, by instantiating frame-consistent knowledge structures, and inviting structurally-consistent inferences. Interestingly, the influence of the metaphorical framing is covert: people do not recognize metaphors as an influential aspect in their decisions. Finally, the influence of metaphor we find is strong: different metaphorical frames created differences in opinion as big or bigger than those between Democrats and Republicans.
